# Interaction of MLE with CLAMP zinc finger is involved in proper MSL proteins binding to chromosomes in *Drosophila*

**DOI:** 10.1098/rsob.230270

**Published:** 2024-03-13

**Authors:** Evgeniya Tikhonova, Anastasia Revel-Muroz, Pavel Georgiev, Oksana Maksimenko

**Affiliations:** ^1^ Center for Precision Genome Editing and Genetic Technologies for Biomedicine, Institute of Gene Biology, Russian Academy of Sciences, 34/5 Vavilov Street, Moscow 119334, Russia; ^2^ Department of the Control of Genetic Processes, Institute of Gene Biology, Russian Academy of Sciences, 34/5 Vavilov Street, Moscow 119334, Russia

**Keywords:** MSL, sex determination, transcription factor, C2H2 proteins, zinc finger domain

## Abstract

The *Drosophila* male-specific lethal (MSL) complex binds to the male X chromosome to activate transcription. It comprises five proteins (MSL1, MSL2, MSL3, male absent on the first (MOF), and maleless (MLE)) and two long noncoding RNAs (lncRNAs; roX1 and roX2). The MLE helicase remodels the roX lncRNAs, enabling the lncRNA-mediated assembly of the *Drosophila* dosage compensation complex. MSL2 is expressed only in males and interacts with the N-terminal zinc finger of the transcription factor chromatin-linked adapter for MSL proteins (CLAMP), which is important for the specific recruitment of the MSL complex to the male X chromosome. Here, we found that MLE's unstructured C-terminal region interacts with the sixth zinc-finger domain of CLAMP. *In vitro*, 4–5 zinc fingers are critical for the specific DNA-binding of CLAMP with GA repeats, which constitute the core motif at the high affinity binding sites for MSL proteins. Deleting the CLAMP binding region in MLE decreases the association of MSL proteins with the male X chromosome and increases male lethality. These results suggest that interactions of unstructured regions in MSL2 and MLE with CLAMP zinc finger domains are important for the specific recruitment of the MSL complex to the male X chromosome.

## Graphical abstract

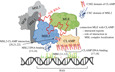

## 1. Introduction

Dosage compensation is a mechanism for compensating gene expression in organisms with an unbalanced number of genes between sexes. In *Drosophila*, sex is determined by the number of X chromosomes: females have two X chromosomes, while males only have one. Dosage compensation is realized via an increase in the expression of male genes on the X chromosome to which the RNA–protein complex that activates transcription binds specifically [1–6]. *Drosophila*'s *d*osage *c*ompensation *c*omplex (DCC) comprises five proteins whose gene mutations are lethal in males. These proteins are called *m*ale-*s*pecific *l*ethal *1* (MSL1), *2* (MSL2), and *3* (MSL3); maleless (MLE); and *m*ale absent *o*n the *f*irst (MOF). The complex also includes two long noncoding RNAs (lncRNAs; roX1 or roX2). MSL1, MSL3, MLE, and MOF are present in both sexes, with only MSL2 and both roX RNAs exclusively expressed in males.

MSL1's C-terminal PEHE (with characteristic amino acidic residues proline (P), glutamic acid (E), histidine (H)) domain is responsible for interacting with MSL3 and MOF [7–10], which together form the functional acetylase module. MSL1's N-terminal coiled-coil domain forms a homodimer that interacts with two MSL2 proteins [7]. It was found that the N-terminal helixes and RING finger (zinc binding domain of C3HC4 involved in ubiquitination) of MSL2 are required for interaction with MSL1 [7]. The MSL1/MSL2 core can specifically bind to a reproducible set of about 200 sites on the X chromosome, which were called the primary chromatin entry (CES) [11] or high-affinity (HAS) sites [12]. A GA-rich sequence motif within the HAS/CES, the MSL recognition element (MRE), is important for DCC targeting [11,12].

**Figure 1.**
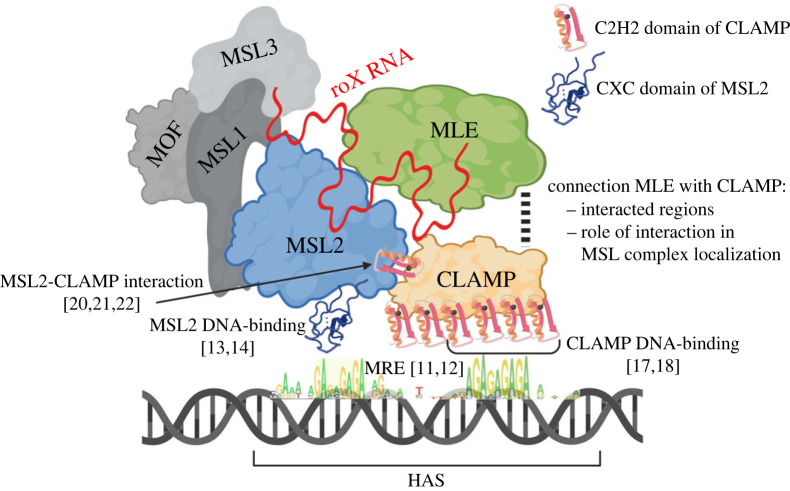
Schematic representation of MSL complex comprising MSL1, MSL2, MSL3, MOF, MLE, CLAMP proteins, and roX lncRNA, recruited to the HAS/CES on X chromosome. MSL2 (via CXC domain) and CLAMP (via 4–7 C2H2 domains) proteins are able to bind with (GA)-rich sequences, which are represented as part of the HAS in MRE. C2H2 domain of CLAMP directly interacts with unstructured region of MSL2. References to relevant works are presented in square brackets.

MSL2 contains the conserved cysteine-rich CXC domain formed by nine cysteines coordinated by three Zn ions (Zn3Cys9) that is the only DNA-binding domain found in the *Drosophila* MSL complex [13,14]. The CXC domain binds with high specificity to MREs *in vitro* [14–16]. Ubiquitous transcription factor *c*hromatin-*l*inked *a*daptor for *M*SL *p*roteins (CLAMP) binds also to GA-rich sequences (MREs) in most of the HAS/CES [17–19] and directly interacts with MSL2's *C*LAMP *b*inding *d*omain (CBD), located distally of its CXC domain [20–22]. It is proposed that the interaction between CLAMP and MSL2 is important for the specific binding of the MSL complex to the male X chromosome [20,21,23]. The CXC domain and CBD of MSL2 are jointly required for the recruitment of MSL to the male X chromosome (figure 1) [13,20,21,23]. However, the question remains how CLAMP specifically interacts with the MSL complex associated with HAS, since there are more than ten thousand CLAMP binding sites on all chromosomes [18].

MLE, an ATP-dependent RNA/DNA helicase of the DEAD subfamily, interacts with the two noncoding roX lncRNAs with high specificity and induces their unwinding [24–30], enabling them to bind to MSL2 [31,32] and potentially other MSL proteins [24,25,32,33]. MSL2's unstructured C-terminal portion is required to integrate the roX lncRNAs into the MSL complex [31,34,35]. Without MSL2, the roX lncRNAs are not included in the MSL1-MSL3-MOF subcomplex [7]. By interacting with MSL proteins, the roX lncRNAs stimulate the assembly of complete active complexes [32–35]. MLE is mainly included in the MSL complex through its interaction with the roX lncRNAs [25,36]. A model has been proposed in which the roX lncRNAs form condensates around the male X chromosome *in vivo* that concentrate MSL proteins, leading to the subsequent specific binding of the MSL complexes to the X chromosome [34,35]. MLE has also been implicated in the spread of the DCC along the X chromosome from the HAS [29,30,37,38]. ChIP-seq binding profiles for all MSL subunits in S2 cells suggest that MSL2 and MLE determine the binding of the MSL complex to HAS [39]. In accordance with the assumption that the protein is involved in the recruitment of the MSL complex to the X chromosome, it was shown that MLE interacts with CLAMP in coimmunoprecipitation from extracts isolated from the CME W1 Cl.8+ cell line [25]. Also the deletion of either the CBD or CXC domain in MSL2 does not significantly affect specific recruiting of MSL on the X chromosome [21] suggesting that other MSL subunits can be responsible for association of the complex with the X chromosome.

The main goal of this study is to elucidate the potential role of MLE in the recruitment of the MSL complex to the X chromosome of males (figure 1). Using the yeast two-hybrid (Y2H) and coimmunoprecipitation assays, we show that MLE's unstructured C-terminal region interacts with CLAMP's six zinc-finger domain. ChIP-seq analysis showed that the deletion of the CBD in MLE affects the recruitment of the MSL complex to the male X chromosome in adult flies.

## 2. Results

### 2.1. MLE interacts with CLAMP's sixth C2H2 domain

**Figure 2.**
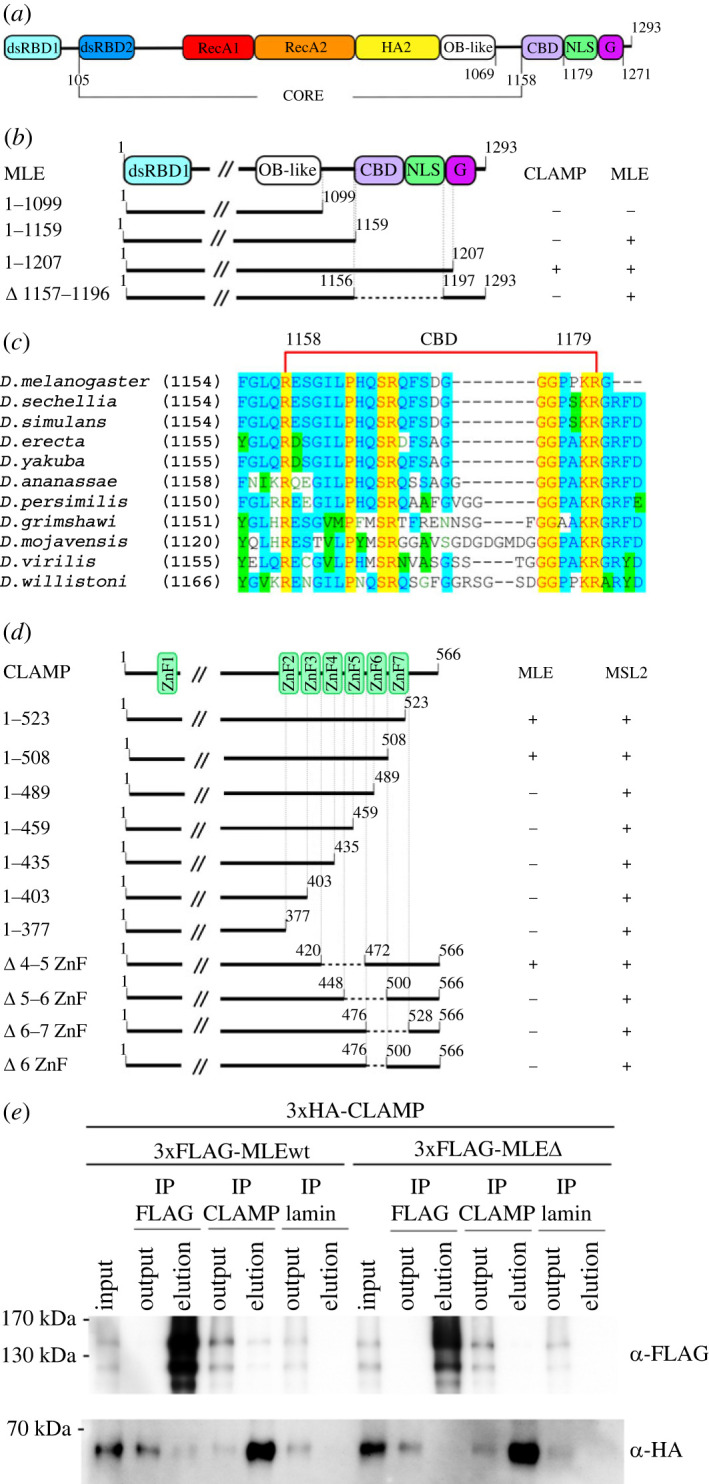
The mapping of interacting regions in CLAMP and MLE. (*a*) Schematic representation of the MLE protein. The dsRBD2, RecA1, RecA2, HA2, and OB-fold domains form the globule core of MLE. CBD, CLAMP binding domain; NLS, nuclear localization signal; G, GGGYGNN heptad repeats. (*b*) The mapping of the MLE domain interacting with CLAMP in the Y2H assay. Different MLE fragments were fused to the GAL4 activating domain (AD) and tested for interactions with CLAMP or MLE fused to the GAL4 DNA-binding domain (DBD). All MLE fragments were tested for the absence of interaction with the GAL4 DBD alone. (*c*) Alignment of the CBD of MLE among Drosophilidae. (*d*) The localization of the CLAMP domain interacting with MLE in the Y2H assay. ZnF, C2H2-type zinc finger domains. Different CLAMP fragments were fused to the GAL4 AD and tested for interactions with MLE or MSL2 fused to the GAL4 DBD. All CLAMP fragments were tested for the absence of interaction with the GAL4 DBD alone. (*e*) Total extracts from *Drosophila* S2 cells co-transfected with 3xHA-CLAMP and 3xFLAG-MLEwt (MLE^WT^) or 3xFLAG-MLEΔ (MLE^ΔCBD^) were immunoprecipitated with antibodies against FLAG, CLAMP or lamin (used as a negative control), and the immunoprecipitates were analysed by immunoblotting for the presence of FLAG- or HA-tagged proteins. Immunoprecipitates after elution are shown concentrated relative to the input by a factor of 5.

The 1293 amino acid (aa) ATP-dependent DEXH box RNA/DNA helicase MLE (figure 2*a*) contains two N-terminal RNA-binding domains (*d*ouble-*s*tranded *R*NA (dsRNA) *b*inding *d*omain 1 (dsRBD1) and 2 (dsRBD2)) [36], two highly conserved RecA domains [40], a *h*elicase *a*ssociated *2* (HA2) domain, and an *o*ligonucleotide-*b*inding (OB)-fold domain. The region between 105 and 1158 aa forms the core of the MLE protein (figure 2*a*). At MLE's C-terminus is an unstructured region followed by imperfect GGGYGNN heptad repeats that may be involved in nonspecific RNA binding [41]. MLE's C-terminal region has been shown to contain a nuclear localization signal (NLS) and have dimerization activity [41,42].

A potential interaction between MLE and CLAMP was demonstrated only via co-immunoprecipitation and might be mediated by MSL2, which directly interacts with CLAMP. Therefore, we first confirmed that MLE and CLAMP interact directly in the Y2H assay (figure 2*b*; electronic supplementary material, figure S1). We also repeatedly demonstrated that MLE can form dimers. Next, we mapped regions in MLE that interact with CLAMP. Several MLE variants with deletions in the C-terminal region were tested for interaction with CLAMP or MLE. We found that the 1159 aa MLE can interact with wild-type (WT) MLE but not CLAMP; the 1099 aa MLE lost both interaction abilities. Available data [40] showed that the region between 1099 and 1159 aa is included in the overall globular structure of the protein along with all other domains; therefore, deletion of the 1099–1158 aa region is highly likely to result in an incorrectly folded MLE protein. The 1207 aa MLE could interact both with CLAMP and MLE. Deleting the 1158–1195 aa region in MLE results in the loss of the interaction with CLAMP but preserves homodimerization activity. According the MLE structure [40], the 1158–1195 aa region is located outside of the globular core. The 1158–1195 aa region contains moderately conserved sequences only among MLEs of *Drosophila* species (figure 2*c*; electronic supplementary material, figure S2) and directly flanks the potential NLS. The MLE region between 1158 and 1195 aa was called the CBD.

Next, we attempted to map the region in CLAMP responsible for its interaction with MLE (figure 2*d*). CLAMP is a 566 aa transcription factor that contains a single C2H2-type N-terminal zinc finger (C2H2 domain) and a C-terminal cluster of six C2H2 domains (figure 2*d*). We previously found that the N-terminal C2H2 domain is required for its interaction with MSL2 [21], while most of the N-terminal intrinsically disordered region (46–86 aa) is responsible for homodimerization [43]. Several C-terminally truncated CLAMP variants were tested for interaction with MLE in the Y2H assay (figure 2*d*; electronic supplementary material, figure S1). The interaction of CLAMP variants with MSL2 was used as a control. The MLE interaction region was mapped to the region of the 6–7 C2H2 domains. Next, we tested interactions for several CLAMP variants with deletion of the 4–5, 5–6, 6–7, and 6 C2H2 domains. Only deleting the sixth C2H2 domain affected the interaction between CLAMP and MLE (figure 2*d*). These results suggest that MLE's unstructured region interacts with the sixth C2H2 domain of CLAMP.

Finally, we confirmed the interaction between the proteins by co-immunoprecipitation of 3×HA-tagged CLAMP and 3xFLAG-tagged MLE^WT^ in co-transfected S2 cells. At the same time 3xFLAG-tagged MLE^ΔCBD^ (deletion of 1158–1179 aa) protein demonstrated the absence of co-precipitation with 3xHA-CLAMP (figure 2*e*). Taken together the results suggest that CLAMP interacts with MLE's unstructured region.

### 2.2. CLAMP's 4–5 C2H2 domains are critical for its specific binding to GA-rich motifs

**Figure 3.**
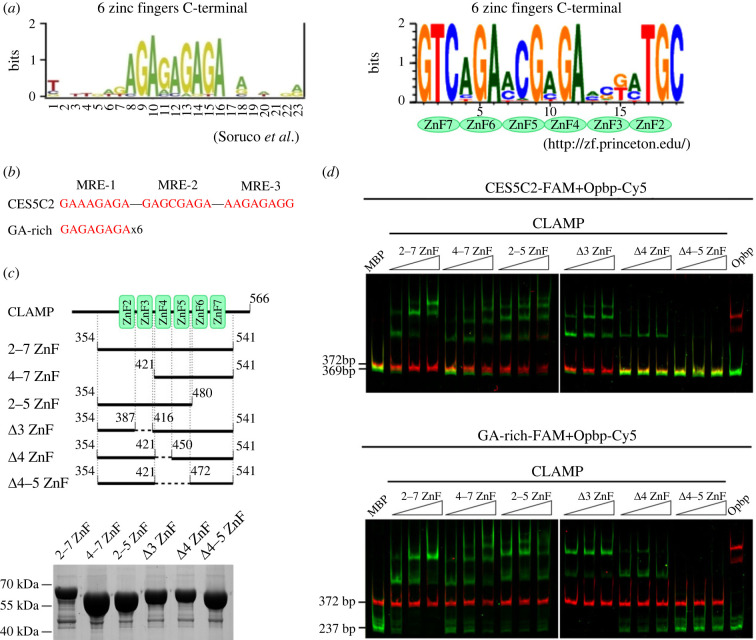
Mapping the C2H2 domains required for CLAMP's specific binding to DNA. (*a*) On the left is a CLAMP motif discovered via protein binding microarrays assessing CLAMP binding to all possible 10-bp sequences [18]. On the right is the predicted binding site for the CLAMP 2–7 C2H2 array (http://zf.princeton.edu; polynomial SVG) [45]. (*b*) The DNA fragments tested for CLAMP binding: 199 bp CES5C2 (with three MRE motifs [11]) and 64 bp (GA)_24_. (*c*) Schematic representation of the CLAMP 2–7 C2H2 zinc finger (ZnF) cluster and its various truncated derivatives that were fused to MBP and expressed in *Escherichia coli*. (*d*) Electrophoretic mobility shift assay of different variants of recombinant zinc finger domains of CLAMP with two DNA fragments. Different zinc finger domains of CLAMP fused with MBP or five zinc finger domains of Opbp fused with MBP or MBP alone were incubated with fluorescently labelled DNA fragments; CLAMP binding sites labelled with FAM (CES5C2-FAM and GA-rich-FAM) and Opbp binding sites (used as a negative control) labelled with Cy5 (Opbp-Cy5). Signals were detected for the FAM-labelled fragment (green bands) at an Ex/Em of 500/535 nm and the Cy5-labeled fragment (red bands) at an Ex/Em of 630/700 nm. Interaction specificity was demonstrated by incubation with DNA fragments (100 ng each) with different amounts of truncated CLAMP variants presented as a series of twofold dilutions (5, 10, and 20 pmol). Note that no binding was observed on the control DNA region (Cy5 signal).

According to a previous study on CLAMP–DNA interaction [18], the sixth domain of C2H2, which interacts with the MLE protein, may also be involved in the DNA binding activity of CLAMP. Therefore, the next task was to map the C2H2 domains that determine the specific CLAMP–DNA interaction. The CLAMP motif was previously discovered via protein binding microarrays assaying CLAMP's binding to all possible 10-bp sequences [18]. CLAMP's 2–7 and 4–7 C2H2 domains bind efficiently to the same (GA)_4_ motif *in vitro* (figure 3*a*). In conventional C2H2 zinc finger proteins, each finger interacts mainly with three adjacent DNA base pairs [44]. The C2H2 domains can be linked in tandem to occupy DNA of varying lengths. The predicted DNA-binding specificity of CLAMP's 2–7 C2H2 domains [45] showed that the 4 and 5 C2H2 domains can jointly bind to the GAGA motif (figure 3*a*), while the 6 C2H2 domain potentially recognizes GA.

To further map the C2H2 domains responsible for binding to the CLAMP motif, we tested different combinations of CLAMP's C2H2 domains for their ability to interact specifically with DNA (figure 3*b*). In this study, we used the 199 bp CES5C2 corresponding to CLAMP's strong binding region, which is essential for recruiting the MSL complex [11,17]. CES5C2 contains three MRE motifs (figure 3*b*). In addition, the (GA)_24_ (64 bp) sequence was used as an alternative CLAMP binding region. Different combinations of C2H2 domains (2–7, 4–7, 2–5, 2–7Δ3, 2–7Δ4, and 2–7Δ4–5) attached to maltose-binding protein (MBP) were expressed in bacteria (figure 3*c*). The electrophoretic mobility shift assay confirmed that both the 2–7 and 4–7 C2H2 domains bind to the tested sequences (figure 3*d*). However, the 2–7 C2H2 domains bind to both tested DNAs with greater affinity. The 2–5 C2H2 domains bound to the DNA fragments more strongly than the 4–7 C2H2 domains. Deleting the single C2H2 domains in the 2–7 C2H2 array showed that the 4 C2H2 domain is essential for specific DNA binding. Deleting the 4 and 5 C2H2 domains eliminated the binding to the DNA fragments. These results are consistent with the prediction [45] (http://zf.princeton.edu), suggesting that the 4–5 C2H2 domains are required for CLAMP's specific binding to chromatin sites. Therefore, CLAMP's 6–7 C2H2 domains are not essential for specific DNA binding and may be involved in its interactions with proteins.

### 2.3. MLE's CLAMP binding domain is required for dosage compensation

**Figure 4.**
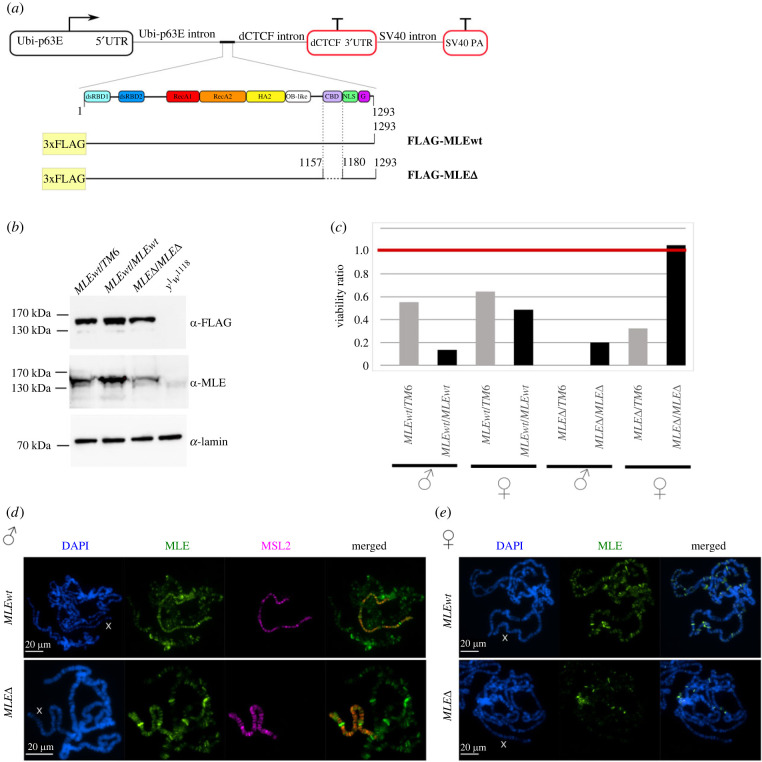
Comparison of the functional activity of MLE^WT^ and MLE^ΔCBD^ *in vivo*. (*a*) Schematic representation of the MLE^WT^-FLAG (MLEwt) and MLE^ΔCBD^-FLAG (MLEΔ) transgenes and proteins expressed in flies. (*b*) Immunoblot analysis of protein extracts prepared from adult flies carrying MLEwt/TM6 (*y^1^w^1118^; mle^9^/mle^9^; Ubi:mle^WT^-FLAG/TM6*); MLEwt/MLEwt (*y^1^w^1118^; mle^9^/mle^9^; Ubi:mle^WT^-FLAG/Ubi:mle^WT^-FLAG*); MLEΔ/MLEΔ (*y^1^w^1118^; mle^9^/mle^9^; Ubi:mle*^Δ*CBD*^*-FLAG/Ubi:mle*^Δ*CBD*^*-FLAG*); *y^1^w^1118^* (control with endogenous MLE). Immunoblot analysis using mouse anti-FLAG, rabbit anti-MLE and mouse anti-lamin Dm0 antibodies (internal control). (*c*) The viability of males and females expressing MLE^WT^-FLAG (MLEwt) and MLE^ΔCBD^-FLAG (MLEΔ) in the *mle^9^*-null background was scored in the progeny of crosses of males and females with genotypes y^1^w^1118^, mle^9^/CyO; Ubi:mle*/TM6, where mle* is MLEwt or MLEΔ. The viability was tested in males and females homozygous (mle*/mle*) or heterozygous (mle*/TM6) for the transgene. The expected viability ratio should be close to 1.0. At least 500 flies were scored in two independent experiments (see electronic supplementary material, table S1). The percentage of obtained females and males with a certain genotype (*y*-axis) was calculated as the ratio of the existing number of flies to the expected number of flies. The expected number of females and males with a particular genotype was calculated based on the best surviving genotype of females (mle^9^/CyO; Ubi:mle*/Ubi:mle*), the viability of which was taken as 100%. (*d*) Distribution of the MSL complex on the polytene chromosomes of male third-day larvae expressing MLEwt (*y^1^w^1118^*; *mle^9^/mle^9^*; *Ubi:mle^WT^/TM6*) and MLEΔ (*y^1^w^1118^*; *mle^9^/mle^9^*; *Ubi:mle*^Δ*CBD*^*/Ubi:mle*^Δ*CBD*^). Panels show protein immunostaining using mouse anti-FLAG (green) and anti-MSL2 (magenta) antibodies. DNA was stained with DAPI (blue). (*e*) Binding of the MLE variants to the polytene chromosomes of female third-day larvae expressing MLEwt and MLEΔ. Panels show protein immunostaining using mouse anti-FLAG antibody (green). DNA was stained with DAPI (blue).

To evaluate the functional effects of deleting the CBD in MLE *in vivo*, we created transgenic flies expressing the MLE variants tagged with the FLAGx3 epitope: MLE^WT^-FLAG (WT) and MLE^ΔCBD^-FLAG (1158–1179 aa deletion). The MLE variants were expressed under the control of a strong ubiquitin-p63E (*Ubi*) promoter (figure 4*a*). In order to avoid the influence of position effects on the expression of MLE^WT^ and MLE^ΔCBD^, both transgenes were inserted into the same genomic location (86Fb) on chromosome 3 using a *φ*C31-based integration system [46].

To test the role of the CBD in the specific recruitment of the MSL complex to the X chromosome in males, we crossed both transgenes into the null *mle* background. In this study, we used the previously characterized loss-of-function mutation in the *mle* gene, *mle^9^* (*mle^y38^*) [47,48]. Next, we compared the expression of MLE^WT^-FLAG and MLE^ΔCBD^-FLAG in 2- to 3-day-old *mle^9^* males (figure 4*b*).

Immunoblot analysis showed that *Ubi:mle^WT^* transgene in a heterozygous state was expressed at a similar level to the *Ubi:mle*^Δ*CBD*^ transgene in a homozygous state. Next we compared expression of the MLE variants in transgenic lines and MLE in the *y^1^w^1118^* line (figure 4*b*). In *mle^9^/mle^9^;Ubi:mle*^Δ*CBD*^*/Ubi:mle*^Δ*CBD*^ and *mle^9^/mle^9^;Ubi:mle^WT^/TM6* males MLE variants are expressed at least twice as strongly as MLE in *y^1^w^1118^* males. As expected in *mle^9^/mle^9^; Ubi:mle^WT^/Ubi:mle^WT^* males MLE are expressed much stronger. We also compared MSL1 and MSL2 expression (electronic supplementary material, figure S3) in *y^1^w^1118^, mle^9^/mle^9^;Ubi:mle^WT^/TM6* and *mle^9^/mle^9^;Ubi:mle*^Δ*CBD*^*/Ubi:mle*^Δ*CBD*^ lines. Immunoblot analysis revealed no significant difference of the expression of MSL1 and MSL2 in these lines.

Like other null *mle* mutants, homozygous *mle^9^* females were viable, while males died during development. We compared the viability of *mle^9^* females expressing either MLE^WT^-FLAG or MLE^ΔCBD^-FLAG (figure 4*c*; electronic supplementary material, table S1). Unexpectedly, strong overexpression of MLE^WT^ (*Ubi:mle^WT^/Ubi:mle^WT^*) affected viability of males and females in the *mle^9^/mle^9^* but not in the *mle^9^/CyO* background. It is likely that the *mle^9^* chromosome contains additional recessive mutations/allelic variants that interfere with MLE overexpression. Lower expression of MLE^WT^ (*Ubi:mle^WT^/TM6*) resulted in almost complete restoration of *mle^9^* male survival. Moreover, *mle^9^/mle^9^; Ubi:mle^WT^/TM6* line is stable, and males and females of this line have approximately the same survival rate (electronic supplementary material, table S2). These results suggest that *Ubi:mle^WT^/TM6* almost completely compensates the *mle^9^* mutation and restores dosage compensation.

In contrast to MLE^WT^, the viability of *mle^9^/mle^9^* flies increased with the expression of the mutant protein MLE^ΔCBD^ in the *Ubi:mle*^Δ*CBD*^ homozygotes (figure 4*c*; electronic supplementary material, table S1). In the *Ubi:mle*^Δ*CBD*^*/TM6* background, only *mle^9^/mle^9^* females are viable. Increasing of the MLE^ΔCBD^ expression in *Ubi:mle*^Δ*CBD*^*/Ubi:mle*^Δ*CBD*^ homozygotes led to better survival of males, which appear in a ratio of one to five females. *mle^9^/mle^9^;Ubi:mle*^Δ*CBD*^*/Ubi:mle*^Δ*CBD*^ males were weak and died within one week after hatching. In addition, we were unable to support the *mle^9^/mle^9^;Ubi:mle*^Δ*CBD*^*/Ubi:mle*^Δ*CBD*^ line, which was lost within a few generations (electronic supplementary material, table S2). Altogether, these results suggest that deleting the CBD in MLE affects dosage compensation in males.

We next examined whether deleting the CBD in MLE affects the recruitment efficiency of the MSL complex to the male X chromosome. Immunostaining of polytene chromosomes from the salivary glands of *Drosophila* larvae allows for the visualization of proteins on interphase chromatin and has been used extensively to study dosage compensation [9,49–53]. Polytene chromosomes had the same MSL1 and MSL2 distribution pattern in *Ubi:mle^WT^* and *Ubi:mle*^Δ*CBD*^ larvae: only the X chromosome was covered by these proteins (figure 4*d*). MLE (detectable by anti-FLAG antibodies) colocalized with MSL2 (figure 4*d*) and MSL1 (electronic supplementary material, figure S4) on the X chromosome but was also found in bands and puffs on all chromosomes. The same MLE binding pattern was observed in females lacking the MSL complex (figure 4*e*). The C-terminal 353 aa of MLE (lacking the helicase domain) has been previously shown to bind to all chromosomes [54]. This association is RNA sensitive, implicating MLE's C-terminal domain in nonspecific interactions with RNA. Taken together, the immunostaining of polytene chromosomes showed that MLE^WT^-FLAG and MLE^ΔCBD^-FLAG bind with the same efficiency to the MSL complex on the X chromosome, confirming that deletion of CBD in the MLE protein does not affect its presence in the MSL complex.

### 2.4. Deletion of CLAMP binding region of MLE affects the recruitment of the MSL complex to the X chromosome in adult males

The results presented in the previous section show that deletion of CBD in the MLE protein significantly reduces the survival of males compared to females, indicating a decrease in the efficiency of dosage compensation. On the other hand, on the polytene chromosomes of larvae we do not observe any effect on the binding of the MSL proteins upon expression of the MLE^ΔCBD^ mutant in comparing with MLE^WT^. It was previously shown that the deletion of CLAMP binding domain in the MSL2 protein also does not affect the binding of the complex to the X chromosome on the polytene chromosomes of male larvae [21]. Therefore ChIP-seq (chromatin immunoprecipitation-sequencing) as a more sensitive approach was used to investigate the potential role of CBD in MLE to recruit the MSL complex to the adult male X chromosome.

**Figure 5.**
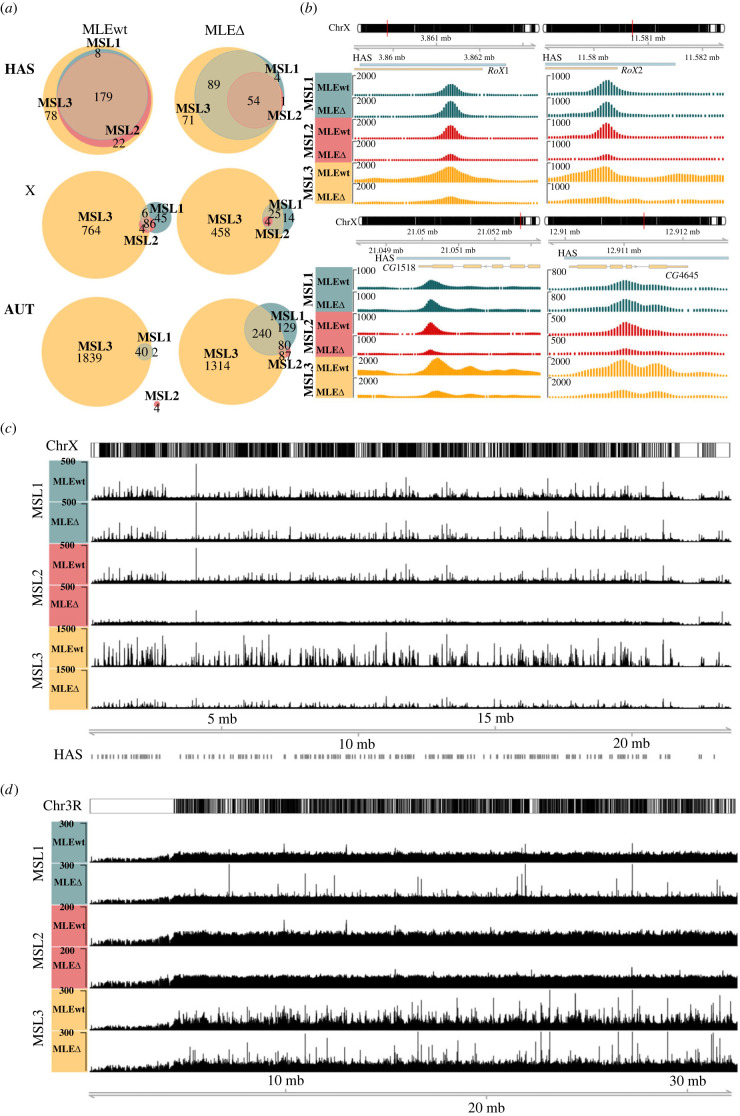
Deletion of the MLE CBD causes chromosome-scale MSL protein redistribution. To study the functional role of MLE's CBD in recruiting the DCC to chromatin sites, we compared the ChIP-Seq profiles of MSL1, MSL2, and MSL3 in three-day males expressing MLEwt (*y^1^w^1118^; mle^9^/mle^9^; Ubi:mle^WT^/TM6*) and MLEΔ (*y^1^w^1118^; mle^9^/mle^9^; Ubi:mle*^Δ*CBD*^*/Ubi:mle*^Δ*CBD*^). (*a*) A Venn diagram of MSL1, MSL2, and MSL3 peak intersections for the MLEwt and MLEΔ lines. All peaks were divided into three groups: coincident with HAS on the X chromosome (HAS), other peaks on the X chromosome (X), and autosomal peaks (AUT). (*b*) Examples of MSL1, MSL2, and MSL3 signal distributions on HAS for *RoX1*, *RoX2*, *CG1518*, and *CG4645* areas. Blue and yellow tracks represent HAS and gene positions, respectively. (*c*) Normalized (reads per kilobase per million mapped reads (RPKM)) signals for the MSL1, MSL2, and MSL3 proteins on chromosome X for the MLEwt and MLEΔ lines. (*d*) Normalized (RPKM) signals for the MSL proteins on chromosome 3R for the MLEwt and MLEΔ lines.

We compared the binding of MSL1, MSL2, and MSL3 in males expressing either MLE^WT^-FLAG (*mle^9^*/*mle^9^; Ubi:mle^WT^/TM6*) or MLE^ΔCBD^-FLAG (*mle^9^*/*mle^9^; Ubi:mle*^Δ*CBD*^*/Ubi:mle*^Δ*CBD*^) (figure 5). We performed ChIP-seq analysis of the chromatin collected from three-day-old flies. For ChIP we used antibodies against the MSL1, MSL2, and MSL3 for both MLE^WT^-FLAG and MLE^ΔCBD^-FLAG transgenic lines, followed by massively parallel sequencing using an Illumina NovaSeq platform. Peaks were identified for MSL1 (294 and 568, respectively), MSL2 (223 and 82), and MSL3 (2948 and 2271) in MLE^WT^-FLAG and MLE^ΔCBD^-FLAG males (electronic supplementary material, table S3). All peaks were divided into three groups: coincident with HAS on the X chromosome, other peaks on the X chromosome (outside the HAS) (X), and autosomal peaks (AUT; figure 5*a*). It is worth noting that the peaks and their signals found for the MLE lines are mainly consistent with the control *y^1^w^1118^* line (electronic supplementary material, figure S5). CLAMP binds to most HAS but is not enriched at autosomal peaks associated with MSL proteins [15,18,20,55].

Notably, MSL2 binding was significantly (Wilcoxon signed-rank test, *p*-value = 3.28 × 10^−23^) weakened in the HAS regions of the MLE^ΔCBD^-FLAG line, with more than half of the peaks disappearing. In addition, there was a detectable 2- to 3-fold reduction in MSL3 binding levels across the X chromosome, with approximately 70% of the original peaks retained. However, MSL1 binding to the X chromosome remained almost unchanged (figure 5*a–c*). Interestingly, MSL1 binding to the autosomes was noticeably enhanced in the MLE^ΔCBD^-FLAG line (figure 5*a*,*d*).

**Figure 6.**
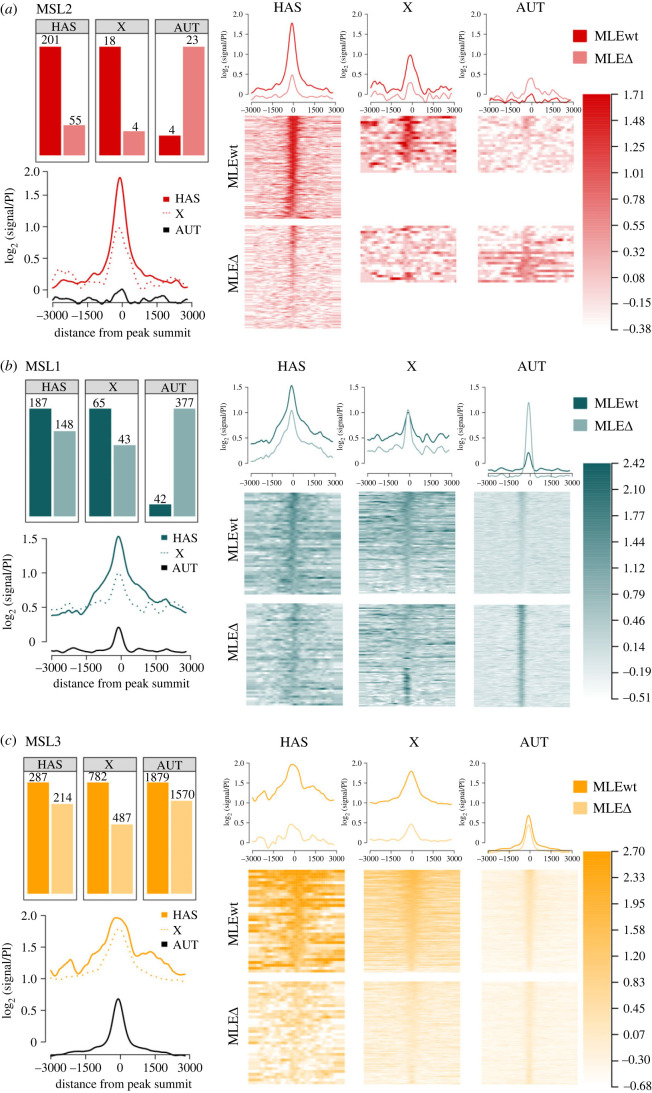
Deleting MLE's CBD alters MSL protein binding. (*a–c*) A histogram of peak counts (top left), and average log fold-change (FC) between normalized (RPKM) signals and nonspecific IgG signals in different genomic regions (HAS, X chromosome, AUTosomes) of the MLEwt line (bottom left). Histograms show average log FC between normalized (RPKM) test signals and nonspecific IgG signals of the MLEwt and the MLEΔ lines in different genomic regions (HAS, X chromosome, AUTosomes) (top right), and heatmaps (bottom right) for MSL2, MSL1, and MSL3, respectively. Peak lists and scales, unified between fly lines, were used to build heatmaps and profiles separately for each MSL protein. Peaks were arranged in descending order of the average signal calculated at vicinity of ±250 bp from the middle of the peak for the MLEwt. LogFC was calculated after smoothing signals using the Daniell kernel with kernel size 100 and adding a pseudo count.

The ChIP-seq data from adult MLE^WT^-FLAG males showed that the MSL proteins bound to approximately 90% of the previously identified HAS from three studies [11,12,56] (figure 6; electronic supplementary material, figure S5). MSL1 and MSL2 bound directly to HAS, while MSL3 was associated with extended regions surrounding the HAS (figure 5*b*).

MSL2 bound predominantly to all HAS on the X chromosome in MLE^WT^-FLAG males (figure 6*a*). MSL2 was partially redistributed from HAS (only 25% remained significantly enriched for MSL2) to a group of autosomal peaks in MLE^ΔCBD^-FLAG males. The average signal for MSL2 was also reduced for the remaining HAS in MLE^ΔCBD^-FLAG males.

Unlike MSL2, the number of peaks on the X chromosome for MSL1 did not differ much between MLE^ΔCBD^-FLAG and MLE^WT^-FLAG males (figure 6*b*). However, approximately 330 new peaks for MSL1 appeared on the autosomes in MLE^ΔCBD^-FLAG males, which predominantly showed promoter localization (electronic supplementary material, figure S6*a*,*b*). The average signal for MSL1 decreased slightly for HAS and remained almost unchanged for other parts of the X chromosome. For the new peaks on the autosomes, we detected significant signal increases (Wilcoxon signed-rank test, *p*-value = 1.77 × 10^−39^) in MLE^ΔCBD^-FLAG flies (figure 6*b*; electronic supplementary material, figure S6*a*,*c*). Therefore, deleting the CBD in MLE causes a redistribution of MSL1 to autosome sites with simultaneous preservation of the general binding profile along the X chromosome.

MSL3 showed peaks at HAS and at an additional 800 sites on the X chromosome and about 1900 sites on autosomes (figure 6*c*). The average signal for MSL3 peaks on the X chromosome was several times larger than for peaks on the autosomes. The number of peaks just slightly decreased in MLE^ΔCBD^-FLAG males. However, the average signal for the X-chromosome peaks was significantly reduced (Wilcoxon signed-rank test, *p*-value = 2.26 × 10^−106^). A comparison of MSL3 profiles on the X chromosome outside of HAS and the autosomes demonstrated the predominant preservation of its binding profile in MLE^ΔCBD^-FLAG flies (figure 6*c*; electronic supplementary material, figure S6). In a large proportion of cases, protein binding regions extend into the body of genes (electronic supplementary material, figure S6*b*). We found a more significant increasing of MSL1 binding in MLE^ΔCBD^-FLAG flies at the peaks with MSL3 colocalization (Wilcoxon signed-rank test, *p*-value = 1.15 × 10^−6^) (electronic supplementary material, figure S6*c*).

Altogether, these results indicate that deleting the CBD in MLE decreases MSL2 and MSL3 binding to HAS on the X chromosome. It also significantly increases MSL1 binding and redistributes MSL3 binding to autosomes in adult males. Taken together these results suggest that the CBD in the MLE protein participates in the process of the recruitment of the MSL complex to the male X chromosome.

## 3. Discussion

Our results provide evidence that MLE's small unstructured region between its main core region and C-terminal NLS [41] interacts with the sixth zinc-finger domain of CLAMP. MLE’s primary function is the assembly of the MSL complex since only MSL1/MSL2 are found at the entry sites in its absence [49,57]. MLE specifically interacts with two roX lncRNAs and induces their unwinding [24–30,58], enabling both to interact with MSL proteins [24,25,31–33]. Interactions between the MSL proteins and roX lncRNAs increase the efficiency of the complex formation. Our Y2H results showed that MLE can dimerize and support a previous finding that the glycine-rich heptad repeats are involved in this process [41]. MLE dimerization may promote the formation of centres for assembling MSL complexes.

Here, we have described a new function of MLE in dosage compensation associated with a specific interaction with CLAMP. Our genetic and ChIP-seq experiments showed that the CBD in MLE contributes to the specific recruitment of MSL complexes to the male X chromosome. The interaction of MLE with CLAMP could increase the specificity of MSL binding to HAS. Previous Chip-seq analysis in S2 cells showed that MLE and MSL2 are located closer to the DNA and thus might be important for recruiting the MSL complex to the HAS [39]. Interestingly, deletion of only the CBD in either MLE or MSL2 has only a weak effect on the specific recruitment of the MSL complex to the X chromosome, which is especially evident from the efficient binding of the complex to the polytene X chromosome of males expressing mutant variants of MLE or MSL2 [21]. This suggests that partially redundant CLAMP–MLE and CLAMP–MSL2 interactions cooperatively improve the stability and efficiency of MSL recruitment to the X chromosome.

The interaction between MLE's unstructured region and the sixth C2H2 domain in CLAMP is a new example of the involvement of C2H2 domains in specific protein–protein interactions. We have previously shown an interaction between MSL2 and the N-terminal domain of CLAMP zinc finger [21,22]. These results demonstrate for the first time that the C2H2 domains of a single architectural protein, such as CLAMP, can simultaneously interact with different unstructured regions of two proteins that form the transcription complex, resulting in more specific and stable recruitment to regulatory elements. Interactions between C2H2 domains in DNA binding proteins and intrinsically disordered regions of subunits of transcription complexes may be widespread for the specific recruitment of transcription complexes to regulatory elements. Despite the key role of C2H2 proteins in the formation of promoter architecture [59,60], the involvement of C2H2 domains in the recruitment of transcription complexes remains poorly understood. It is likely that identifying other protein–protein interactions that determine the specificity of recruitment of the MSL complex to the X chromosome will allow us to better understand the contribution of C2H2 domains to this process.

We found that only the 4–5 С2Н2 domains are critical for CLAMP's binding to DNA motifs *in vitro*, while the 3, 6, and 7 C2H2 domains improve binding effectiveness. Since MSL2's CXC domain also binds to GA repeats [13,14], CLAMP likely binds to part of the GA repeats in HAS. We hypothesize that CLAMP binds to HAS/CES in cooperation with additional DNA-binding proteins that together form a chromatin module leaving CLAMP's zinc fingers 1 and 6 free to interact with the MSL complex. At autosomal sites, CLAMP presumably binds in cooperation with alternative protein groups, which form chromatin sites in which interactions with proteins or DNA mask its 1 and 6 C2H2 domains. For example, in embryos CLAMP functions together with a key pioneer transcription factor Zelda that also contains C2H2 domains [61]. The model can explain the recent results that ectopic expression of MSL2 in females results in preferential binding of MSL2 to autosomal promoters enriched in TGTG motifs (1093) rather than GAGA motifs and CLAMP sites (363) [62]. Since CLAMP binds to more than 10 000 sites on autosomes [18], it seems likely that the N-terminal zinc finger domain of CLAMP is masked to interact with MSL2 at autosomal sites.

CLAMP's 6 and 7 C2H2 domains are highly conserved in insects. In contrast, the region of MLE corresponding to its CBD is only conserved in the *Drosophila* genus. Moreover, even in species remote from *Drosophila melanogaster*, like *Drosophila virilis*, there are many substitutions and insertions in this region of MLE. Similarly, the CBD in MSL2 is also only conserved in the *Drosophila* genus [21,34]. It seems likely that unstructured regions in MSL proteins can rapidly evolve to form new specific interactions with the zinc finger domains of DNA-binding proteins, allowing the specific interactions between DNA-bound architectural proteins and transcriptional complexes to be organized and improved.

## 4. Material and methods

### 4.1. Plasmid construction

Plasmids for the yeast two-hybrid assay were prepared using the full-sized and truncated versions with separate domains of MLE and CLAMP as C-termini fused with pGBT9 and pGAD424 vectors from Clontech. Different full-sized variants of MLE were fused with 3xFLAG at the N-terminus and cloned into an expression vector. This vector contains *attB* site for *φC31*-mediated recombination, *Ubi63E* promoter with its 5'UTR, 3′UTR with SV40 polyadenylation signal, intronless *yellow* gene as a reporter for detection of transformants. Details of the cloning procedures, primers and plasmids used for plasmid construction are available upon request.

### 4.2. Yeast two-hybrid assay

Yeast two-hybrid assay was carried out using yeast strain pJ69-4A, with plasmids and protocols from Clontech. For growth assays, plasmids were transformed into yeast strain pJ69-4A by the lithium acetate method, as described by the manufacturer, and plated on media without tryptophan and leucine. After 2 days of growth at 30°C, the cells were plated on selective media without tryptophan, leucine, histidine and adenine, and their growth was compared after 2–3 days. Each assay was repeated three times.

### 4.3. Antibodies

Antibodies against MSL1[423-1030], MSL2[421-540], CLAMP and MSL3 were raised in rabbits and purified from the sera by ammonium sulfate fractionation followed by affinity purification on CNBr-activated Sepharose (GE Healthcare, USA) or Aminolink Resin (ThermoFisher Scientific, USA) according to standard protocols. Other antibodies: mouse monoclonal anti-FLAG, clone M2 (F1804, Sigma, USA), mouse monoclonal anti-HA, clone HA-7 (H3663, Sigma, USA), mouse monoclonal anti-lamin Dm0, clone ADL84.12 (ADL84.12, DSHB, USA).

### 4.4. Co-immunoprecipitation assay

*Drosophila* S2 cells were co-transfected by 3xHA-CLAMP and 3xFLAG-MLE^WT^ or 3xFLAG-MLE^ΔCBD^ plasmids with MACSFectin (Miltenyi Biotec, USA). After transfection, the cells were incubated for 48 h and then collected by centrifugation at 700*g* for 5 min, washed once with 1×PBS, and resuspended in 20 packed cell volumes of hypotonic lysis buffer (20 mM Tris–HCl, pH 7.4, with 10 mM KCl, 10 mM MgCl_2_, 2 mM EDTA, 10% glycerol, 1% Triton X-100, 1 mM DTT, 1 mM PMSF and Calbiochem Complete Protease Inhibitor Cocktail V). After incubation on ice for 10 min, the cells were sonicated (Bioruptor (Diagenode, USA) for 2 min on setting L, 15 s ON/45 s OFF), NaCl was added to a final concentration of 420 mM, and incubation on ice continued for 60 min, with periodic mixing. Sonication was repeated as above to reduce viscosity, cell debris was pelleted by centrifugation at 10 000*g* for 30 min at 4°C, and the supernatant was collected for immunoprecipitation with anti-FLAG-, anti-lamin-conjugated Protein G Magnetic beads, and anti-CLAMP-conjugated Protein A Magnetic beads (NEB, USA) (by incubating in the PBST on a rotary shaker at room temperature for 1 h) equilibrated in incubation buffer-150 (20 mM Tris–HCl, pH 7.4, with 150 mM NaCl, 10 mM MgCl_2_, 1 mM EDTA, 1 mM EGTA, 10% glycerol, and 0.1% NP-40). The protein extract (50 µg protein) was adjusted to a volume of 500 µl with buffer-150, mixed with antibody-conjugated beads (30 μl), and incubated on a rotary shaker overnight at 4°C. The beads were then washed with five portions of buffer-150, and resuspended in SDS-PAGE loading buffer, boiled and analysed by immunoblot analysis. Proteins were detected using the ECL Plus Western Blotting substrate (Thermo Fisher Scientific, USA).

### 4.5. Fly crosses and transgenic lines

*Drosophila* strains were grown at 25°C under standard culture conditions. The transgenic constructs were injected into preblastoderm embryos using the *φC31*-mediated site-specific integration system at locus 86Fb [46]. The emerging adults were crossed with the *y^1^ ac w^1118^* flies, and the progeny carrying the transgene in the 86Fb region were identified by *y^+^* pigmented cuticle. Details of the crosses and primers used for genetic analysis are available upon request.

### 4.6. Fly extract preparation

Twenty adult flies were homogenized with a pestle in 200 µl of 1×PBS containing 1% β-mercaptoethanol, 10 mM PMSF and 1 : 100 Calbiochem Complete Protease Inhibitor Cocktail VII. Suspension was sonicated 3 times for 5 s at 5 W. Then, 200 µl of 4×SDS-PAGE sample buffer was added and the mixture was incubated for 10 min at 100°C and centrifuged at 16 000*g* for 10 min.

### 4.7. Electrophoretic mobility shift assay

Aliquots of purified recombinant proteins were incubated with fluorescently labelled DNA fragments in the presence of nonspecific binding competitor poly(dI-dC). Incubation was performed in PBS (pH 8.0) containing 5 mM MgCl_2_, 0.1 mM ZnSO_4_, 1 mM DTT, 0.1% NP-40 and 10% glycerol at room temperature for 30 min. The mixtures were then resolved by nondenaturing 5% PAGE (79 AA:1 BAA) in 0.5 × TBE buffer at 5 V cm^−1^. Signals were detected for FAM-labelled fragment at the Ex 500 nm/Em 535 nm and for Cy5-labelled fragment at the Ex 630 nm/Em 700 nm.

### 4.8. Immunostaining of polytene chromosomes

*Drosophila* 3rd instar larvae were cultured at 18°C under standard conditions. Polytene chromosome staining was performed as described [21]. The following primary antibodies were used: rabbit anti-MSL1 at 1 : 500 dilution, rabbit anti-MSL2 at 1 : 500 dilution, and monoclonal mouse anti-FLAG at 1 : 100 dilution. The secondary antibodies were Alexa Fluor 488 goat anti-mouse 1 : 2000 and Alexa Fluor 555 goat anti-rabbit 1 : 2000 (Invitrogen). The polytene chromosomes were co-stained with DAPI (AppliChem). Images were acquired on a Nikon Eclipse Ti fluorescent microscope using a Nikon DS-Qi2 digital camera, processed with ImageJ 1.50c4 and Fiji bundle 2.0.0-rc-46. Three to four independent stainings and 4–5 samples of polytene chromosomes were performed with each MLE-expressing transgenic line.

### 4.9. ChIP-Seq

Chromatin was prepared from two- to three-day-old adult males. One gram of adult flies was ground in a mortar in liquid nitrogen and resuspended in 20 ml of buffer A (15 mM HEPES–KOH, pH 7.6, 60 mM KCl, 15 mM NaCl, 13 mM EDTA, 0.1 mM EGTA, 0.15 mM spermine, 0.5 mM spermidine, 0.5% NP-40, and 0.5 mM DTT) supplemented with 0.5 mM PMSF and Calbiochem Complete Protease Inhibitor Cocktail V. The suspension was then homogenized in a Potter and Dounce homogenizer with a tight pestle, filtered through a 100 µm nylon cell strainer (Miltenyi Biotec, USA), and cross-linked with 1% formaldehyde for 15 min at room temperature. Cross-linking was stopped by adding glycine to a final concentration of 125 mM. The nuclei were washed with three 10 ml portions of wash buffer (15 mM HEPES–KOH, pH 7.6, 60 mM KCl, 15 mM NaCl, 1 mM EDTA, 0.1 mM EGTA, 0.1% NP-40, and protease inhibitors) and one 5 ml portion of basic nuclear lysis basic buffer (15 mM HEPES, pH 7.6, 140 mM NaCl, 1 mM EDTA, 0.1 mM EGTA, 1% Triton X-100, 0.5 mM DTT, 0.1% sodium deoxycholate, and protease inhibitors) and resuspended in 1 ml of nuclear lysis buffer (15 mM HEPES, pH 7.6, 140 mM NaCl, 1 mM EDTA, 0.1 mM EGTA, 1% Triton X-100, 0.5 mM DTT, 0.1% sodium deoxycholate, 0.5% SLS, 0.1% SDS, and protease inhibitors). The suspension was sonicated in a Covaris ME220 focused-ultrasonicator (40 alternating 15-s ON and 45-s OFF intervals, peak power 75, duty % factor 25), and 50 µl aliquots were used to test the extent of sonication and measure the DNA concentration. Debris was removed by centrifugation at 14 000*g*, 4°C, for 10 min, and chromatin was pre-cleared with Protein A Dynabeads (Invitrogen, USA). Corresponding antibodies were incubated for 1 hour at room temperature with 20 µl aliquots of Protein A (anti-MSL1, 1 : 100; anti-MSL2, 1 : 100; anti-MSL3, 1 : 500) Dynabeads (Invitrogen, USA) mixed with 200 µl of PBST. Then antibody–Dynabead complexes were washed and equilibrated in nuclear lysis buffer. Chromatin samples containing 10–20 µg of DNA equivalent in 200 µl nuclear lysis buffer (2 µl aliquots of pre-cleared chromatin as input material) were incubated overnight at 4°C with antibody–Dynabead complexes. After 3 rounds of washing with lysis buffer supplemented with 500 mM NaCl and TE buffer (10 mM Tris–HCl, pH 8; 1 mM EDTA), the DNA was eluted with elution buffer (50 mM Tris–HCl, pH 8.0; 1 mM EDTA, 1% SDS), the cross-links were reversed, and the precipitated DNA was extracted using a ChIP DNA Clean &Concentrator kit (Zymo Research, USA).

The ChIP-seq libraries were prepared with NEBNext Ultra II DNA Library Prep kit per the manufacturer's instructions. Amplified libraries were quantified using fluorometry with DS-11 (DeNovix, USA) and Bioanalyzer 2100 (Agilent, USA). Diluted libraries were clustered on a pair-read flowcell and sequenced using a NovaSeq 6000 system (Illumina, USA).

ChIP-seq analysis was performed on paired-end reads data. Each sample was presented in 2 biological replicas. Preprocessing and peak calling was performed based on the pipeline described previously [63]. The cutadapt [64], Bowtie2 [65] and MACS2 [66] were employed for trimming, mapping and peak calling respectively. dm6 version of *Drosophila melanogaster* genome was used as a reference genome. Upon merging the replicates, coverage tracks in BedGraph format were generated using the deepTools [67] bamCoverage function, with a bin width of 50 bp, and extendReads option applied. The coverage tracks were further normalized by reads per kilobase of transcript, per million mapped reads (RPKM).

We assessed reproducibility using the IDR pipeline (https://sites.google.com/site/anshulkundaje/projects/idr), with *p-*value thresholds for true replicates, pseudoreplicates, and MACS2 was selected 0.05 and 0.01 respectively achieving rescue ratio (RR) or self-consistency ratio (SR) being less than 2.

Further analysis was conducted using R version 4.2.1 (http://www.r-project.org). To track changes in the ChIP-seq signal level between different lines, a unified list of peaks was created for each protein, using the findOverlapsOfPeaks function from ChIPpeakAnno package [68]. The average signal was calculated using getTagMatrix from ChIPseeker package [69]. Part of the peaks were assigned to HAS, by intersecting them with a list of combined HAS variants from three different studies [11,12,56]. Additionally closest to peaks genomic features were annotated using the annotatePeak function from clusterprofiler [70] with tssRegion set to 200. Gviz [71] were used for genomic track visualization. To measure the significance level of the signal change between MLE^ΔCBD^ and MLE^WT^ fly lines, we conducted Wilcoxon signed-rank tests on groups of the average signals calculated at vicinity of ±1000 bp from the middle of the peaks.

## Data Availability

Raw and processed ChIP-seq data are deposited in the NCBI Gene Expression Omnibus (GEO) under accession number GSE239354. Additional information is provided in electronic supplementary material [72].
